# TP53 mutations in vulval lichen sclerosus adjacent to squamous cell carcinoma of the vulva

**DOI:** 10.1038/sj.bjc.6601444

**Published:** 2003-12-09

**Authors:** K J Rolfe, A B MacLean, J C Crow, E Benjamin, W M N Reid, C W Perrett

**Affiliations:** 1 University Department of Obstetrics & Gynaecology, Royal Free and University College Medical School, Royal Free Campus, Rowland Hill Street, London NW3 2PF, UK; 2 Histopathology Department, Royal Free and University College Medical School, Royal Free Campus, Rowland Hill Street, London NW3 2PF, UK; 3 Department of Histopathology, Royal Free and University College Medical School, Bloomsbury Campus, London WC1E 6HX, UK

**Keywords:** immunohistochemistry, lichen sclerosus, mutation, p53, squamous cell carcinoma of the vulva

## Abstract

Non-neoplastic epithelial lesions of the vulva (NNEDV) lichen sclerosus (LS) and squamous hyperplasia (SH) have been implicated in the pathogenesis of squamous cell carcinoma of the vulva (SCC). To date, there have been no recognisable precursor lesions for SCC associated with NNEDV. TP53 is the most frequent genetic change in human cancers and can indicate both aetiology and molecular pathogenesis of tumours. A total of 27 SCC patients underwent immunohistochemistry (IHC) and TP53 mutational analysis using microdissection and direct sequencing. There were 19 patients with areas of adjacent epidermis: 17 had NNEDV (four SCCs had more than one adjacent lesion) and two had normal epidermis. In all, 70.4% of the SCCs, 40% LS and 22.2% SH demonstrated overexpression of p53. In total, 77.8% of SCCs, 46.7% of LS and 22.2% SH demonstrated mutations in TP53, with the majority of lesions having a mutation in codon 136. Eight cases were identified where the same mutation was identified in the SCC and in the adjacent area. These data suggest that TP53 mutations develop in NNEDV and are intrinsic to the clonal evolution that leads to SCC. The type of mutation detected is more likely to occur due to endogenous cellular changes rather than exogenous carcinogen exposure.

Lichen sclerosus (LS) is an inflammatory disease of unknown aetiology and pathogenesis. It is a disorder of the skin, which is most common in the genital area but can occur anywhere on the body. It affects both sexes and all age groups. In a review by Meffert *et al* (1995) of 5207 patients, the female-to-male incidence was reported as 6 : 1, with genital involvement in 83% of cases. The majority of sufferers of anogenital LS are either middle-aged or elderly women. The predominant symptom in females is an intractable itch, which is often associated with dysuria, dyspareunia, dryness of the skin, labial stenosis or fusion and, in children, constipation (Laude *et al*, 1980).

There have been many studies assessing the risk of squamous cell carcinoma of the vulva (SCC) in inflammatory diseases of the vulva. Wallace (1971) reported that 12 of 290 (4%) patients developed SCC following a history of LS during a 12-year period but further follow-up studies have shown a wide range of risk of progression (Hart *et al*, 1975; Meffert *et al*, 1995; Carlson *et al*, 1998). Others (Rueda *et al*, 1994; Scurry *et al*, 1997; Vilmer *et al*, 2000) have looked at the skin adjacent to SCC which, not uncommonly, shows epithelial disorders; the most common are LS and squamous hyperplasia (SH).

The TP53 gene is located on chromosome 17p13.1; it consists of 11 exons with 10 introns. Exon 1 is noncoding, while exons 5–8 are part of the highly evolutionarily conserved domain. Mutations in the TP53 gene are the most frequent genetic changes in human cancers, and the spectrum of mutations can indicate tumour aetiology and molecular pathogenesis (Levine *et al*, 1991; Greenblatt *et al*, 1994). In addition, a comparison of the mutation profile between malignant and potential premalignant lesions can give an indication of the clonality and progression of such tumours.

The aim of this study was to assess the presence of TP53 mutations in SCC and the adjacent non-neoplastic epithelial disorders of the vulva (LS and SH; NNEDV) and to compare the results of the mutation analysis with immunohistochemsitry (IHC) results. It was hoped that the data would also provide information on the clonality of SCC and the carcinogenic progression of adjacent lesions.

## MATERIALS AND METHODS

### Sample details

A total of 27 formalin-fixed, paraffin wax-embedded vulvectomy specimens from the files of the Histopathology Departments of the Royal Free and University College Medical School (Royal Free and Gower Street Campuses) were used with a mean age of 71.6±8.6 years (range 49–86 years). Nineteen patients also had adjacent epidermis available for microdissection; 17 of these had an NNEDV (LS and/or SH) with a total of 24 NNEDV specimens and two had normal epidermis adjacent to the SCC.

### Immunohistochemical analysis

Sections (4 *μ*m) were cut for immunohistochemical analysis and histopathological confirmation of diagnosis by two pathologists (EB, JCC).

The IHC procedure has been described previously (Rolfe *et al*, 2001,^2002^). Briefly, sections were dewaxed in xylene and rehydrated through different grades of ethanol up to distilled water. Endogenous peroxidase was blocked by using a 3% hydrogen peroxide solution. Antigen retrieval was performed by heating the sections in sodium citrate buffer in a microwave (2 × 5 min). Nonspecific binding was blocked by preincubation with 10% normal rabbit serum for 10 min at room temperature. A primary mouse monoclonal antibody, which recognises both wild-type and mutant p53 (DO-7, Dako, Ely, Cambs, UK) was applied at 1 : 100 dilution for 1 h at room temperature. All stages were carried out at room temperature with phosphate-buffered saline used for washing between stages. Following incubation with a secondary antibody, a standard streptavidin–biotin–horseradish complex was then used, with 3, 3′-diaminobenzidine tetrahydrochloride as the chromogen to detect the presence of the p53 protein. Slides were counterstained with Mayers' haemalum (Merck, Lutterworth, Leics, UK).

To ensure staining consistency and reproducibility, a number of precautions were taken: (i) All sections from different blocks were stained as a batch on the same day and the process was repeated four times for each protein; and (ii) results were analysed independently by the three observers (EB, JCC and KJR). In all cases, there was <5% variation in staining between sections and observers.

p53 staining was classified as the percentage of positive (brown) nuclei in specific epidermal areas or as a percentage of tumour cells with ⩽10% counted as negative and >10% as positive, as described previously (Rolfe *et al*, 2002).

### Microdissection

**Figure 1:**
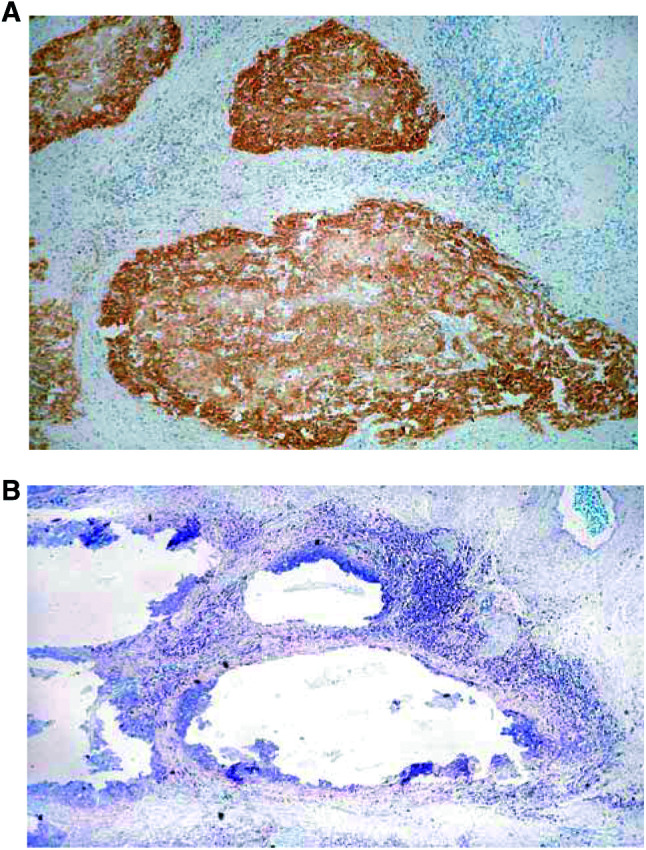
(**A**) A slide stained by IHC showing overexpression of p53 in the SCC. (**B**) A toluidine blue-stained adjacent slide demonstrating the area that had shown overexpression of p53, removed by microdissection.

Two sections sequential to the IHC stained slides were dewaxed and rehydrated up to sterile, double-distilled water. Sections were then stained with toluidine blue (Sigma-Aldrich, Poole, Dorset, UK) which enabled the various lesions to be easily identified. Lesions were carefully removed without contamination from surrounding tissue (Figure 1A, B

) using a sterile technique with a 27-gauge needles (Davies Health Care) and each lesion placed separately into separate sterile Eppendorf microcentrifuge tubes. Lesions were flooded with 200 *μ*l of digestion buffer (10 mM Tris-HCl, pH 8.5, 1 mM EDTA, 0.5% Tween 20®) containing 1 mg ml^−1^ of proteinase K (Sigma-Aldrich, Poole, Dorset, UK). Samples were incubated at 37°C for 20 h and then heated to 95°C for 10 min to inactivate the proteinase K. The solution was then centrifuged at 3500 revolutions per minute for 10 min and the supernatant removed, placed in a sterile 1.5 ml Eppendorf tube, and stored at 4°C.

### PCR

PCR was performed using commercially available primers for exons 5–8 of TP53 (Cruachem, Glasgow, Scotland; Table 1

**Table 1:** Primers used for TP53 PCR amplification

Exon 5	Forward	GGAATTCTGTTCACTTGTGCCCTGACTTTCAAC
	Reverse	GCAACCAGCCCTGTCGTCTCTCCA
Exon 6	Forward	CCAGGCCTCTGATTCCTCACTGATTG
	Reverse	AGGGCCACTGACAACCACCCTTAAC
Exon 7	Forward	CCTGCTTGCCACAGGTCT
	Reverse	CCGGAAATGTGATGAGAGGT
Exon 8	Forward	AGGTAGGACCTGATTTCCTTACTGCC
	Reverse	GGAATTCTGAGGCATAACTGCACCCTTGGTCT

). PCR was performed using 4 *μ*l of the DNA extracted from the paraffin sections in a 50 *μ*l PCR mix of 1.5 mM MgCl_2_, 14–17 pmoles of each primer, 200 *μ*M deoxynucleoside triphosphates (Pharmacia, Milton Keynes, Bucks, UK), in the PCR buffer (20 mM (NH_4_)_2_SO_4_, 75 mM Tris-HCl, pH 8.8, 0.01% Tween®) with 2.5 U *Taq* enzyme (‘Thermus icelandicus’; Advanced Biotechnologies, Epsom, Surrey, UK). PCR was performed over 35 cycles with an annealing temperature for the four exons of 60°C. PCR products, along with a negative control (DNA omitted and sterile double-distilled water used instead) were run on a 10% nondenaturing polyacrylamide gels to assess for the presence of product and any contamination.

### Sequencing

Sequencing was performed commercially from both strands of the DNA (MWG-Biotech, Milton Keynes, Bucks, UK). Briefly, this involves reamplification of the product using nested primers, followed by M13 sub-cloning. Detection was by a Li-Cor infrared detection system. When a mutation was detected, the PCR and the sequencing were both repeated to confirm the result.

### Statisticss

A *P*-value of <0.05 was considered to be statistically significant and *χ*^2^ analysis was used.

## RESULTS

### p53 IHC

**Figure 2:**
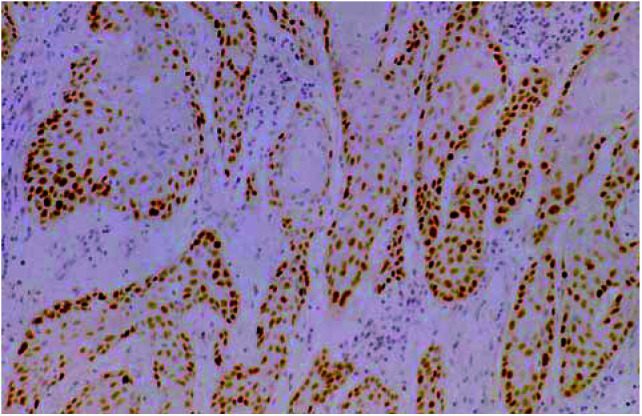
Overexpression of p53 in a well-differentiated SCC: magnification × 40.

In total, 70.4% (19 out of 27) of SCCs demonstrated overexpression of p53 (>10% nuclear staining). Squamous cell carcinoma of the vulva showed different patterns of staining depending on the grade of the tumour with well-differentiated tumours demonstrating expression around the periphery of the keratin pearls and moderately to poorly differentiated SCCs demonstrating expression throughout the tumour (Figure 2

). Results are summarised in Table 2

**Table 2:** Results of immunohistochemistry and mutational analysis

	**Lesion**	**Immunohistochemistry**	**Mutation**
1	SCC	+	Exon 5 codon 136
	SH	+	Exon 5 codon 136
	LS	+	Exon 5 codon 136
2	SCC	+	Exon 5 codon 136
	N	−	Wild type
3	SCC	−	Exons 5 & 7, codons 136 and 244
4	SCC	+	Exon 5, codon 136
	N	−	Wild type
5	SCC	+	Exon 5, codon 136
	SH	−	Wild type
6	SCC	−	Wild type
	SH	−	Wild type
7	SCC	+	Wild type
8	SCC	+	Exon 5, codon 136
	LS	−	Exon 5, codon 136
	SH	−	Wild type
	LS^a^	−	Wild type
9	SCC	−	Exon 5, codon 136
10	SCC	+	Exon 5, codon 136
	LS	−	Exon 5, codon 136
11	SCC	−	Exon 5, codon 136
	SH	−	Wild type
	LS	−	Exon 5, codon 136
12	SCC	+	Exon 5, codon 136
	SH	−	Wild type
13	SCC	+	Exon 5 & 7, codons 136 & 248
	LS	+	Exon 5 & 7, codons 136 & 248
14	SCC	+	Wild type
	LS	+	Wild type
15	SCC	+	Exon 6, codon 213
	LS	−	Wild type
16	SCC	+	Exon 7, Intron between 7 & 8
17	SCC	−	Exon 5, codon 136
	SH	−	Exon 5 &7, codons 136 & 248
18	SCC	+	Exon 5, codon 136
	LS	−	Wild type
19	SCC	−	Exon 5 & 7, codon 136 & 258
	SH	−	Exon 7, codon 258
20	SCC	−	Exon 5 & 7, codon 136 & 235
	LS	−	Exon 5, codon 136
21	SCC	+	Exon 5 & 7, codons 136 & 234
22	SCC	−	Exon 6, codon 214
	LS	−	Wild type
23	SCC	+	Exon 5, codon 136
	LS	+	Exon 5, codon 136
24	SCC	+	Wild type
25	SCC	+	Exon 5 & 7, codon 136 & 248
26	SCC	+	Exon 5, codon 136
27	LS^b^	−	Wild type
	LS^b^	+	Wild type
	SCC	+	Wild type
	LS	+	Wild type
	SH	+	Wild type

SCC=squamous cell carcinoma of the vulva; LS=lichen sclerosus; SH=squamous hyperplasia.

a Lesion not adjacent to SCC.

b Lesions taken at different time.

.

None of the normal epidermis adjacent to SCC demonstrated overexpression of p53. In all, 40% (six out of 15) of the LS lesions did demonstrate overexpression of the p53 protein, and staining was confined to the basal/parabasal layers of LS. In all 22.2% (two out of nine) of adjacent SH areas were identified as overexpressing p53 with staining identified throughout the epidermis.

There was no statistically significant difference demonstrated between any of the lesions when using *χ*^2^ analysis.

### TP53 mutations

**Figure 3:**
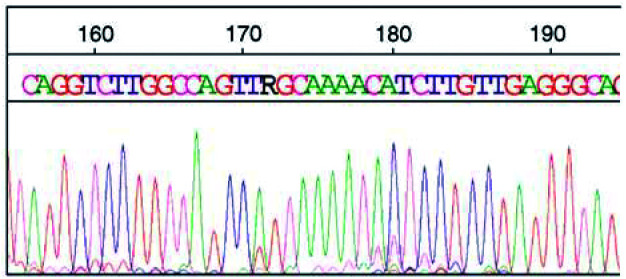
The TP53 sequence for part of the reverse strand of exon 5. The R demonstrates a heterozygous mutation in codon 136. This sequence was produced from LS adjacent to SCC. The green peak indicates an A, which in wt TP53 is a G (small red peak). This sequence was confirmed by analysis of the forward strand and repeat PCR and sequencing.

In total, 77.8% (21 out of 27) of SCCs demonstrated a TP53 mutation (in codons 136, 213, 214, 234, 235, 248); two SCCs demonstrated mutations and polymorphisms (exons 244 and 258) with 19 SCCs demonstrating the same mutation in exon 5 (codon 136:**C**AA → **T**AA, Gln → stop; Figure 3

) and a further SCC demonstrating a mutation in the intron between exons 7 and 8. In all, 46.7% (seven out of 15) of adjacent LS lesions were identified as containing a mutation in the TP53 gene with all identified as having the mutation in codon 136; however, one LS lesion had the mutation in codon 136 with a further mutation in exon 7 (codon 248:C**G**G → C**A**G; Arg → Gln). In all, 22.2% (two out of 9) of adjacent SH were identified as having a mutation in the TP53 gene with all (mutant positives) identified as having the mutation in codon 136 and one SH lesion identified as having the mutation in codon 136 plus a mutation in exon 7, codon 248.

### Clonality analysis

Eight cases were identified where the same mutation was identified in the SCC and the adjacent lesion (with a further adjacent lesion demonstrating the same polymorphism as the SCC) resulting in 50% (eight out of 16) of our SCCs having an adjacent lesion with the same clone of cells. When studying individual lesions, 46.7% (seven out of 15) of adjacent LS lesions and 33.3% of adjacent SH showed the same clone of cells as the adjacent SCC.

### Correlation between mutation and IHC

In all, 66.7% (14 out of 21) of SCC, which overexpressed p53, demonstrated a mutation, though p53 overexpression was also identified in SCCs that showed wild-type TP53 (83.3%). Adjacent lesions showed correlation with wild-type TP53 and no overexpression of p53 (Table 3

**Table 3:** Correlation of immunohistochemistry and mutations in each lesion

**Lesion**	**IHC+/mt+**	**IHC−/mt+**	**IHC+/mt−**	**IHC−/mt−**
SCC	66.7% (14/21)	33.3% (7/21)	83% (5/6)*	16.7% (1/6)
SH	50% (1/2)	50% (1/2)**	14.3% (1/7)	85.7% (6/7)
LS	42.8% (3/7)	57% (4/7)	37.5% (3/8)	62.5% (5/8)

* Mutation in intron considered wild-type TP53.

** Polymorphism considered wild-type TP53. IHC=immounohistochemistry; mt=mutation; SCC=squamous cell carcinoma of the vulva; SH=squamous hyperplasia; LS=lichen sclerosus.

).

## DISCUSSION

### Correlation of sequencing and IHC data

There was a correlation (66.7%) between TP53 mutations and p53 protein overexpression in SCC, but positive IHC expression did not necessarily imply a mutation with 83% of SCCs overexpressing p53 having wild-type TP53.

The antibody used, DO-7, is known to attach to the amino terminus of the human p53 protein at amino-acid location 21–25 (Vojtesek *et al*, 1992; Stephen *et al*, 1995) and is able to detect the overexpression of both wild-type and mutant p53. The antibody detects a stabilised form of the protein that, if not stabilised through mutation, is stabilised through other means, which results in an interruption of the degradative pathway of wild-type p53 (Wynford-Thomas, 1992).

### TP53 mutations in SCC

In total, 77.8% of the SCCs in this study showed a mutation in the TP53 gene. It is possible that those SCCs where no TP53 mutation was identified could have mutations outside exons 5–8 of the TP53 gene, though it has been estimated that less than 5% of mutations occur outside this highly conserved area.

This work has demonstrated a higher percentage of SCCs with a TP53 mutation compared with that found by other authors, who have shown a range of 32–58% (Kim *et al*, 1996; Sliutz *et al*, 1997; Ngan *et al*, 1999). There are two possible reasons for this difference: (i) patient details; the majority in this group were older than the other studies and the majority were associated with NNEDV; and (ii) technical differences with this study using direct sequencing and microdissection.

### TP53 mutation profile in SCC

The mutation profile in the TP53 gene can indicate the potential cause of the mutation. Three codons (213, 244 and 248) from this work were from CpG sites and other authors have shown that such CpG sites are important regions for mutation in SCC (Milde-Langosch *et al*, 1995; Pilotti *et al*, 1995; Kim *et al*, 1996; Ngan *et al*, 1999). Transitions at CpG sites are believed to be caused by endogenous processes. There are many endogenous processes that could result in carcinogenesis, and these have in part been attributed to cell proliferation (Ames and Gold, 1990). Tissue regeneration after toxicity could increase the frequency of mutation from replication errors. Other processes, such as inflammation, oxygen radical damage accompanying tissue injury, and a flux of macrophages may contribute to the mutation frequency (Harris *et al*, 1978).

It is possible that the profound itching and resultant scratching associated with NNEDV may contribute to the pathogenesis of associated SCC. It has previously been demonstrated that there is increased cell proliferation in LS and LS adjacent to SCC (Rolfe *et al*, 2001,^2002^) and there may therefore be an increased likelihood of a spontaneous mutation (Hietanen *et al*, 1995). Inflammatory processes may also be involved in oxidative damage caused by free radicals (Carlson *et al*, 1998). The inflammatory infiltrate of LS has been shown to possess in excess of 10% of macrophages (Tsang and Chan, 1992; Carlson *et al*, 2000), which, if activated, are capable of producing free radicals that have been shown to have a mutagenic activity (Weitzman and Stossel, 1981; Weitberg *et al*, 1983).

The mutation at codon 136 was demonstrated in half of all lesions studied, and results in a **C**AA → **T**AA, that is, amino-acid change gln → stop. Up to 1996, there had been 10 reports of this mutation in sporadic tumours (Hollstein *et al*, 1996), including squamous cell carcinoma of the head and neck (Chung *et al*, 1993), actinic keratosis (Ziegler *et al*, 1994) and other cancers, for example, oesophageal carcinomas (Huang *et al*, 1993).

### TP53 mutations in adjacent lesions

Alterations in the p53 pathway are generally regarded as occurring prior to invasion of the basement membrane in tumours of the lung, oesophagus, head and neck, breast and cervix, and as a late event (i.e. after invasion has occurred) in ovarian cancer. In ulcerative colitis and other inflammatory conditions, TP53 mutations have been found in the earliest recognised dysplastic lesions (Burmer *et al*, 1991,^1992^; Yin *et al*, 1993). This current work on SCC has indicated that TP53 mutations do appear to be early events with eight patients showing an identical TP53 genotype in the adjacent lesion as in the SCC. The relatively low percentage of mutations in SH (22%) could be due to more contaminating cells being present, for example, inflammatory cells in the thickened epidermis, even with the use of microdissection. Ren *et al* (1996) described the epidermis as being composed of epidermal proliferative units each derived from a single stem cell. It is these stem cells that probably harbour the mutations, which are then passed on to the daughter cells. It has been previously proposed that the stepwise progression of replacement of normal squamous epidermis by atypical keratinocytes does not occur in SCC associated with NNEDV; rather the atypical features are confined to the basal and parabasal layers. Hence, SCC arising from LS may arise directly from basal keratinocytes, which invade the underlying dermis (Carlson *et al*, 1998).

### Clonality in SCC

In total, 50% of patients were identified as showing identical mutations in the SCC and adjacent lesion. Only one other study has identified mutations in LS adjacent to SCC and this was in only one case (Milde-Langosch *et al*, 1995). In our work, 46.7% of adjacent LS and 33.3% of SH adjacent to SCC showed identical mutations to the adjacent SCC. Our data suggest that TP53 mutations develop in NNEDV and are intrinsic to the clonal evolution that leads to SCC, as the multiplicity of TP53 mutational events indicate that identical mutations are unlikely to occur independently (Greenblatt *et al*, 1994).

TP53 mutations appear to play an important role in the pathogenesis of NNEDV to SCC, and those mutations appear to occur through endogenous processes and NNEDV appear to be clonal in origin when comparing TP53 mutational analysis.
